# MRI signs of carotid plaque inflammation in patients with unstable angina: a possible sign of widespread plaque activity

**DOI:** 10.1186/1532-429X-11-S1-P21

**Published:** 2009-01-28

**Authors:** Carlo Liguori, Luigi Natale, Agostino Meduri, Antonio Bernardini, Antonella Lombardo, Lorenzo Bonomo

**Affiliations:** 1 Università Cattolica del Sacro Cuore – Policlinico A. Gemelli, Rome, Italy; 2 "G. Mazzini" Hospital – ASL Teramo, Teramo, Italy

**Keywords:** Acute Coronary Syndrome, Arterial Wall, Systemic Inflammation, Unstable Angina, Carotid Plaque

## Purpose

Inflammation may contribute to destabilize vulnerable plaques in acute coronary syndromes by promoting rupture and erosion; systemic inflammatory factors could be related with widespread plaque activity in many vascular districts.

The purpose of this study was to evaluate with contrast enhanced-MRI(CE-MRI) plaque inflammation signs in carotid arteries of pts with unstable angina (UA) and to relate them to serum levels of C-reactive protein (CRP), a marker of systemic inflammation.

## Methods

27 pts with carotid plaques, 16 with UA and 11 with stable angina(SA) underwent carotid arteries MRI with SE, FSE and bb-FSE sequences, before and after Gd iv. administration (0.2 mmol/Kg). We considered 3 markers: 1) wall thickening, index of arterial wall edema or infiltration; 2) increased T2 or FSE-STIR signal intensity (SI), index of arterial wall or plaque edema; 3) arterial wall or plaque enhancement, index of increased capillary permeability. Totally 37 plaques were evaluated (17 patients with monolateral and 10 with bilateral stenosis). CRP levels were determined with ELISA essay.

## Results

19 plaques showed wall thickening and/or increased T2 and/or FSE-STIR SI and Gd enhancement, 5 plaques showed only contrast enhancement, whereas 13 plaques had no inflammation signs. CRP levels of patients with enhanced plaques were significantly higher than those of patients without enhancement (median values: 12.5 vs 3.3, p < 0.05).

## Conclusion

Pts with UA showed plaque inflammation signs more frequently than controls; patients with UA and inflammation signs showed higher CRP levels than pts with UA and no inflammation signs. These results suggest a widespread plaque activity, possibly mediated by systemic inflammation, in acute coronary syndromes.

